# Biomaterials with Antibacterial and Osteoinductive Properties to Repair Infected Bone Defects

**DOI:** 10.3390/ijms17030334

**Published:** 2016-03-03

**Authors:** Haiping Lu, Yi Liu, Jing Guo, Huiling Wu, Jingxiao Wang, Gang Wu

**Affiliations:** 1 School of Stomatology, Zhejiang Chinese Medical University, Hangzhou 310053, China; noprint2000@hotmail.com (H.L.); tracyguo1218@hotmail.com (J.G.); 2 Department of Oral Implantology and Prosthetic Dentistry, Academic Center for Dentistry Amsterdam (ACTA), University of Amsterdam and VU University Amsterdam, MOVE Research Institute, Amsterdam 1081LA, The Netherlands; liuyizzd@163.com (Y.L.); g.wu@acta.nl (G.W.); 3 The First Affiliated Hospital, Medical School, Zhejiang University, Hangzhou 310003, China; 4 The First Affiliated Hospital, Wenzhou Medical University, Wenzhou 325000, China

**Keywords:** co-delivery, antibacterial, osteoinductive, antibiotics, bone morphogenetic protein2 (BMP2), infected bone defect, bone regeneration

## Abstract

The repair of infected bone defects is still challenging in the fields of orthopedics, oral implantology and maxillofacial surgery. In these cases, the self-healing capacity of bone tissue can be significantly compromised by the large size of bone defects and the potential/active bacterial activity. Infected bone defects are conventionally treated by a systemic/local administration of antibiotics to control infection and a subsequent implantation of bone grafts, such as autografts and allografts. However, these treatment options are time-consuming and usually yield less optimal efficacy. To approach these problems, novel biomaterials with both antibacterial and osteoinductive properties have been developed. The antibacterial property can be conferred by antibiotics and other novel antibacterial biomaterials, such as silver nanoparticles. Bone morphogenetic proteins are used to functionalize the biomaterials with a potent osteoinductive property. By manipulating the carrying modes and release kinetics, these biomaterials are optimized to maximize their antibacterial and osteoinductive functions with minimized cytotoxicity. The findings, in the past decade, have shown a very promising application potential of the novel biomaterials with the dual functions in treating infected bone defects. In this review, we will summarize the current knowledge of novel biomaterials with both antibacterial and osteoinductive properties.

## 1. Introduction

### 1.1. Infected Bone Defects

Newly regenerated bone tissue with an adequate volume is indispensable to restore the maxillofacial aesthetics and musculoskeletal functions. Large bone defects can occur as the consequence of injury, cancer and inflammation, *etc.* The repairing rate of a bone defect mainly depends on the size of a bone defect [1]. When the size is greater than the healing capacity of bone tissues, the fibrous connective tissues, that can migrate faster than osteoblasts, dominantly occupy the bone defects [1,2,3,4]. A critical-sized bone defect is the prototype of discontinuity defects, with a failed spontaneous repair [1,2].

The self-repairing capacity of bone can be further limited when a bacterial activity occurs in bone defects. Infected bone defects can be resulted from acute high-energy injuries and chronic infectious diseases. According to the World Health Organization, injuries are a global public health problem [5]. There are approximately 3–9 million injuries recorded annually in developed countries. With the development of economy and transportation, road traffic-related high-energy injuries account for nearly 1.2 million deaths. The number of deaths due to injuries has doubled in the last 30 years in European countries [6]. High-energy injuries to the extremities can result in open comminuted fractures. This kind of injuries are often associated with severe soft tissue damages, bone defects, infections, and ultimately nonunion. High-energy injuries can easily develop to severe osteomyelitis when a bacterial infection is introduced due to a compromised immune protection. Osteomyelitis is difficult to completely eliminate and remains a formidable medical problem, particularly when it accompanies with large bone defects. *Staphylococcus aureus* is the main bacterial pathogen since it is accounted for 80% of human osteomyelitis [7].

Another major cause of infected bone defects is chronic bacterial infections, such as periodontitis and peri-implantitis. Periodontitis and peri-implantitis are the inflammatory diseases surrounding natural teeth and dental implants with the loss of supporting bone through a bacterial etiology. The prevalence of severe periodontitis with deep periodontal pockets (≥6 mm) ranges from 10% to 15% in adults [8]. The incidence rate of peri-implantitis is 8.6% to 9.7% after 5 years [9,10] and as high as 14% after 10 to 15 years [11]. Peri-implantitis exhibits a more pronounced inflammatory progress in mucosa than periodontitis [12]. In addition, the inflammatory lesion of peri-implantitis extends more rapidly into bone marrow and hence progresses a greater extent of bone loss than periodontitis [13,14]. Peri-implantitis can lead to the failure of implants and impose health and financial burdens on patients and health providers. Both diseases have similar pathological bacteria, such as the red complex species (e.g., *Porphyromonas gingivalis* and *Tannerella forsythia*) and orange complex species (e.g., *Prevotella intermedia*) as well as *Aggregatibacter actinomycetemcomitans* [15].

### 1.2. Current Clinical Treatments

Standard treatments for infected bone defects include the removal of necrotic bone fragments, a local and/or systemic administration of antibiotics, and a reconstruction of the bone defects by bone grafts. However, these treatments are really time-consuming (often takes several months to years) and are not always yielding satisfactory outcomes. This situation is largely due to the difficulties in controlling infections, to the delay in bone reconstruction tissues (which is usually undertaken only after the complete eradication of infections), and to the slow progress of new bone formation. Our recent study indicated that the bacteria adherent to an implant could become a potential source of infection of the surrounding tissue [16]. Consequently, it is in great need of an effective method that can simultaneously eradicate infection and promote new bone formation.

In addition to the delivery of antibiotics, a system that treats infected bone defects requires special properties, which includes osteoconductive and osteoinductive properties. Autografts are still regarded as the “gold standard” bone grafts because they possess all the important osteogenic elements: osteoconductive scaffolds, osteogenic cells and osteoinductive growth factors [17]. However, the autografting is associated with a series of limitations, such as the pain and morbidity in donor sites [18,19], a prolonged surgery, a limited available volume [20] and uncontrollable resorption rates [21,22]. The conventional alternative to an autograft is an allograft [23,24]. Although it provides a good, natural and bony scaffold, allogeneic bone grafts are associated with certain risks, such as disease transmissions [25], sterilization-associated toxicities [26], variable host immune responses [27] and limited supplies [17]. As alternatives to autologous and allogeneic grafts, inorganic and organic matrices (e.g., deproteinized bovine bone [28] and demineralized bone collagen [29]) or synthetic polymers (e.g., PLGA (co-polymer of lactic and glycolic acid)) are of unlimited available volumes and have been widely accepted for bone reconstructions. However, none of them is intrinsically osteoinductive to induce *de novo* bone formation in pro-fibrotic microenvironments, such as critical-size bone defects.

### 1.3. Novel Biomaterials with Both Antibacterial and Osteoinductive Properties

In the past decade, novel biomaterials, bearing both antibacterial and osteoinductive properties, have been developed in order to provide a viable treatment option for infected bone defects [30,31,32]. Many novel antibacterial drugs, such as nanosilver/silver nanoparticles (AgNPs), quanternized chitosan, show a very promising clinical application potential due to their good biocompatibility and a broad bactericidal spectrum [33,34]. On the other hand, many growth factors, such as basic fibroblast growth factor, insulin-like growth factors (IGFs), and vascular endothelial growth factor, are also capable of significantly promoting osteogenesis and angiogenesis [35]. However, only bone morphogenetic proteins (BMPs) can uniquely induce *de novo* bone formation in a pro-fibrotic microenvironment, such as critical-size bone defects [36]. Thanks to the rapid progress of bone tissue engineering techniques, a large variety of novel BMPs-based bone-filling materials have been developed to significantly accelerate and promote new bone regeneration. In this review, we will summarize the current knowledge of the novel biomaterials with both antibacterial and osteoinductive properties to treat infected bone defects.

## 2. Osteoinductive Agents and Osteogenic Activities

### 2.1. BMPs

BMPs, a group of proteinaceous growth factors, were discovered in the pioneering work by Urist in 1965 [37]. BMP family consists of more than 30 members [38], among which 19 BMPs are found in human. According to their gene homology, protein structure and functions, the 19 BMPs are further divided into 7 subgroups: BMP2/4, BMP3/3b, BMP5/6/7/8/8b, BMP9/10, BMP11/GDF8, BMP12/13/14 and BMP15/GDF9 [39,40]. Most of the mature BMP molecules (except for GDF3, 9, 9B [41,42]) are comprised of two monomers that are covalently linked with a disulphide bond [40]. The BMP ligand is termed as “BMP homodimer” or “homodimeric BMP” if the two monomers come from the same BMP member. If the two monomers come from different BMP members, the BMP ligand is termed as “heterodimeric BMP” or “BMP heterodimer” [36].

### 2.2. The Signaling Pathways and Osteoinductivity of BMPs

**Figure 1:**
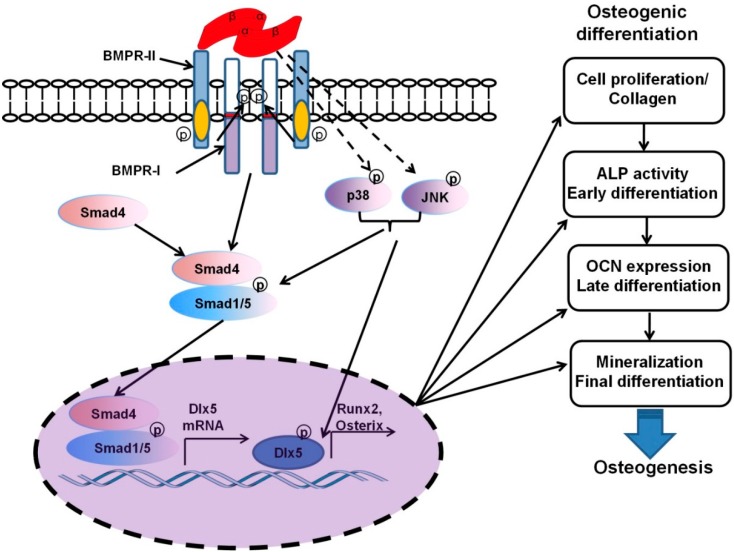
Schematic graphs depicting the signaling pathways of bone morphogenetic proteins (BMPs) and its-induced osteogenic activities. BMPR-I: BMP type I receptors; BMPR-II: BMP type II receptors; Runx2: runt-related transcription factor 2; ALP: alkaline phosphatase; OCN: osteocalcin; Dlx5: distal-less homeobox 5; JNK: c-Jun N-terminal kinase; 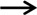: clarified mechanisms; 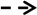: unclarified mechanisms.

The classical role for BMPs was the induction of *de novo* cartilage and bone formation in non-osseous sites [37,43]. BMPs are currently recognized as a group of metabologens that can orchestrate tissue architecture throughout the body [44]. BMPs can significantly promote the differentiation of multipotent mesenchymal stem cells (MSCs) along different lineages, e.g., osteogenesis [45], adipogenesis [46] and chondrogenesis [47]. BMPs bind to their type I and type II transmembrane serine/threonine kinase receptors, thereby initiating their intracellular signaling pathways [48]. The canonical signaling pathway of BMPs is Smad-dependent. Smad1/5/8 can be phosphorylated by the activated BMP receptors and form a complex with Smad4. This complex will then translocate to the nucleus, regulating the transcription of target genes, such as inhibitor of DNA binding 1, distal-less homeobox 5 (Dlx5). Dlx5 plays an important role in upregulating runt-related transcription factor 2 (Runx2) and osterix for osteogenic differentiation (Figure 1). In addition, BMPs can also trigger many Smad-independent signaling pathways, such as mitogen-activated protein kinase pathways [including p38, extracellular signal-related kinase and c-Jun N-terminal kinase (JNK)]. These pathways have essential roles in BMP-induced osteogenic events [49]. During the process of osteoblastogenesis, MSCs will proliferate and undergo differentiation that is symbolized by the expression of alkaline phosphatase (ALP, a marker for the early osteogenic differentiation) and osteocalcin (OCN, a marker for the late differentiation). The final differentiation is symbolized by mineralization of extracellular matrix [50].

### 2.3. Clinical Applications of BMPs

In USA, Europe and Australia, BMP2 and BMP7 have already been approved for a clinical application in nonunion bone fractures and spinal fusions [40]. In current clinic practice, BMP2 is typically used through its superficial adsorption onto collagen sponges (e.g., INFUSE^®^). Deproteinized bovine bone was also rendered osteoinductive using the same principle [51]. However, this out-of-date carrying mode of BMP2, that was developed decades ago, shows a series of potential side effects, such as an over-stimulated osteoclastic bone resorption and an excessive ossification at unintended sites [52,53,54]. Owing to the continuous efforts, a sustainable and physiological delivery mode has been widely accepted to be critical to maximize the osteoinductive efficacy of BMPs [55,56,57].

### 2.4. BMPs with a Higher Osteoinductive Efficiency

BMP2 and BMP-7 are the most widely used BMPs to confer potent osteoinductivity to orthopaedic implants. Recently, we showed that BMP2/7 could more rapidly induce the *in vitro* osteoblastogenesis of pre-osteoblasts in a significantly higher dose-efficiency than homodimeric BMP2 or BMP7 [50]. Moreover, heterodimeric BMPs could more rapidly facilitate the *in vivo* bone formation in a peri-implant bone defect with a more mature microarchitecture than homodimeric BMPs [58]. Other studies also showed that BMP9 was advantageous over BMP2 and BMP7 in inducing the osteogenesis of MSCs [59,60]. These findings suggested that heterodimeric BMPs and BMP9 had a very promising potential in clinical application.

## 3. Antimicrobials

For the treatment of infected bone defects, antibiotics, such as tetracycline and vancomycin are conventionally used in clinic [61]. However, the treatments with systemic antibiotics alone suffer from several drawbacks, such as a potential systemic toxicity and poor penetration into necrotic tissues at wound sites [62]. To approach these problems, many locally delivering systems have been developed [62]. On the other hand, antibiotics usually bear a narrow antibacterial spectrum. A long-term local administration of antibiotics may also cause antibiotic-resistance. In these cases, a series of novel antibacterial biomaterials, such as AgNPs and quanternized chitosan show a very promising application potential since they have a broader antibacterial spectrum and bear nearly no resistance.

### 3.1. Antibiotics

#### 3.1.1. Tetracyclines

Tetracyclines are of the polyketide class and are primarily bacteriostatic antimicrobials. They exert their antibacterial activity by inhibiting the synthesis of microbial proteins [63]. Tetracyclines are produced by the *Streptomyces genus* of *Actinobacteria* and have a broad-spectrum antibacterial activity. Originally, most of medically relevant bacteria were proven to be sensitive to teracyclines. However, the versatility of tetracyclines has been greatly compromised by the fierce increase of acquired resistance in many pathogenic organisms, such as *Staphylococcus* spp., *Streptococcus* spp., *Neisseria gonorrhoeae* and *Enterobacteriaceae*. Tetracyclines, including tetracycline, minocycline, and doxycycline were widely used as topical therapy to treat periodontitis and peri-implantitis [64,65]. In addition, tetracyclines were also used during bone grafting procedures because of their anticollagenase, antibacterial and fibroblast-stimulatory properties [66,67,68]. Tetracycline, minocycline, and doxycline have been incorporated into gels, chips, polymeric fibers and microcapsules to accomplish a sustained level of antibiotics at the site of infections, such as peri-implantitis.

#### 3.1.2. Vancomycin

Vancomycin, an *amycolatopsis orientalis*-derived peptide [69] is usually used as a last resort medication for the treatment of serious, life-threatening infections by gram-positive bacteria that are unresponsive to other antibiotics [70]. However, Gram-negative bacteria are insensitive to vancomycin. Vancomycin takes its effect through inhibiting proper cell wall synthesis.

#### 3.1.3. Tobramycin

Tobramycin, a *Streptomyces tenebrarius*-derived aminoglycoside antibiotic, is used to treat various bacterial infections that are particularly caused by Gram-negative bacteria. *Pseudomonas* is especially sensitive to tobramycin. Tobramycin takes effects by preventing the formation of the 70S complex [71]. As a result, mRNA cannot be translated into protein, which leads to a cell death. Tobramycin has a narrow spectrum of activity [72]. It is not active against Gram-positive bacteria, except for *Staphylococcus aureus*.

#### 3.1.4. Effect of Antibiotics on *in Vitro* Osteogenic Activities

Park *et al.* evaluated the effects of tetracyclines, minocyclines and doxyclines at the concentrations of 10 or 100 μM on the osteogenic activities of murine calvarial pre-osteoblast cells (MC3T3-E1 cell line) [73]. The morphology and viability of the cells were significantly affected by all the selected antibiotics at 100 μM as well as 10 μM tetracycline hydrochloride. Except for 10 μM doxycline hydrochloride, all the antibiotics resulted in significantly lower ALP activities than the control. For the final mineralization, a significant reduction was observed for the treatments of all the selected antibiotics of 100 μM as well as 10 μM tetracycline hydrochloride. Western blot analysis showed that tetracycline, minocycline, and doxycline reduced the endogenous expression of BMP2 and estrogen-α. These data showed that higher levels of tetracycline could result in a dose-dependent decrease of osteogenic protein expression and cell differentiation. Doxycline hydrocloride treatment seemed to give a less negative effect on osteoblasts. Consequently, it might be assumed that it could enable more regenerative healing [74]. In a latter report, the author investigated the effects of low-dose (0.1 μM) doxycyline on the osteogenic differentiation of MC3T3-E1 cells [75]. The ALP activity significantly increased in the presence of 0.1 and 1 μM of doxycline hydrochloride, which might be due to the significantly upregulated expression of estrogen-α. The author concluded that a low dose of doxycline could favor the osteogenic differentiation at an early stage [75]. Interestingly, in another report, although 1 μg/mL doxycycline alone could significantly enhance ALP activity and several osteogenic genes, such as osteopotin, osteonectin and osteocalcin, doxycycline could significantly counteract BMP-induced osteogenic activities in periodontal ligament cells [76]. These results suggested that, for the biomaterials with osteoinductive and antibacterial properties, it is not sufficient to only check the effect of antibiotics/antibacterial materials alone on osteogenic activities. Their effects on BMP-induced osteogenic activities should always be investigated.

### 3.2. Antimicrobial Biomaterials

#### 3.2.1. AgNPs

AgNPs are clusters of silver atoms with diameters ranging from 1 to 100 nm. AgNPs have become highly interesting for medical applications because of their antimicrobial, anti-inflammatory, biocompatible and wound-healing-favoring properties [77]. In comparison with the high toxicity of silver ions, AgNPs bear larger surface area-to-volume ratios, greater efficacy against bacteria [78] and, most importantly, a lower toxicity to humans [79]. The minimal inhibitory concentration for AgNPs was 0.7 ng/mL for *Saccharomyces cerevisiae*, 0.35 ng/mL for *Escherichia coli*, and 3.5 ng/mL for *Staphylococcus aureus* [80]. AgNPs can be obtained by donating electrons to the silver ions (Ag^+^) in different reduction systems, such as sodium borohydride and UV light [77]. The reaction conditions are very critical to control the diameter of AgNPs and to prevent their excessive agglomeration. The powerful antimicrobial activities of AgNPs can be attributed to the following orchestrated mechanisms: (1) the damage of bacterial membranes; (2) the inhibition of DNA replications, protein synthesis and enzymatic activity and (3) the alteration of cell respiration [33]. In comparison with traditional antibiotics, AgNPs bear an antibacterial activity of a broader spectrum. Furthermore, there is extremely rare bacterial resistance to AgNPs [81], which suggests the presence of multiple bactericidal mechanisms acting in synergy. This property even confers AgNPs the capacity to remove the biofilm formed by antibiotic-resistant bacteria, such as methicillin-resistant *Staphylococcus aureus* [82]. The antibacterial activity of AgNPs is highly diameter-dependent [83]: Smaller diameters (<30 nm) have been shown to be optimal [33]. In addition, AgNPs have also another advantageous property over antibiotics: an antiviral effect. Such an effect was, at least partially, mediated by preventing gp120 from binding to CD4 [84]. AgNPs have also shown a strong anti-inflammatory effect, which is mediated by reducing the release of inflammatory cytokines [85], decreasing lymphocyte and mast cell infiltration [86] and inducing apoptosis in inflammatory cells [87,88]. AgNPs have already been successfully used in several medical applications: cardiovascular implants [89], central venous catheters [90], neurosurgical catheters [91], bone cements [92] and wound dressings [93]. However, caution should also be taken since AgNPs may also be cytotoxic through its interaction with mitochondria [94] and the induction of apoptosis pathways [95].

The effect of AgNPs on osteogenic activities is highly dependent on its dosage, incubation time, and physical properties. The diameter of AgNPs was shown to be negatively correlated with both cytotoxicity and antibacterial efficacy [94,96]. However, Hussain *et al.* revealed a positive correlation between size and cytotoxicity, which suggested that the cytotoxic effects might also be dependent on other factors, such as the composition of AgNPs [97]. A significant cytotoxicity of Ag^+^ could occur as early as 1 day, the cytotoxicity of AgNPs, whereas, occurred only after incubation for 21 days. Such a time-dependency might be due to the increasing accumulation of AgNPs inside the cells [98]. On the other hand, when applied in suitable concentration, AgNPs could promote osteogenic activities. Qin *et al.* investigated the effects of AgNPs on the osteogenic activities of urine-derived MSCs [99]. They found that AgNPs ≤4 μg/mL didn’t significantly influence the cell viability. The safe concentration of AgNO_3_ was ≤2 μg/mL. Thereafter, they compared the effects of 2 μg/mL AgNO_3_ and 4 μg/mL AgNPs on the osteogenic differentiation makers, such as ALP activity, mineralization and osteogenesis-related genes. The authors found that 4 μg/mL AgNPs were associated with a significantly higher ALP activity and matrix mineralization in comparison with the controls, while 2 μg/mL AgNO_3_ didn’t show such an effect. Compared with the control group (no AgNPs or AgNO_3_), mRNA expression of Runx2, ALP, BMP2, Collagen 1A1, OCN, and osteopontin in AgNPs-exposed cells on days 7, 14, and 21 was increased by: 2.4-fold, 2.0-fold, and 2.3-fold; 5.9-fold, 9.0-fold, and 3.5-fold; 6.6-fold, 11.0-fold, and 2.0-fold; 2.1-fold, 2.0-fold, and 1.8-fold; 4,0-fold, 7.0-fold, and 2.0-fold; and 4.2-fold, 5.2-fold and 2.4-fold, respectively. In contrast, such an effect was not visible for AgNO_3_. Compared with the controls, cells treated with AgNPs showed prominent, well organized, actin stress fibers, suggesting that AgNPs of this diameter could enhance actin polymerization of urine-derived MSCs. However, there were no significant difference in this regard between the cells treated with AgNO_3_ and the control cells [99]. In consistency with these findings, Mahmood *et al.* found that several nanomaterials containing AgNPs could promote mineralization in MC3T3-E1 cells by enhancing the expression of osteogenic genes [100].

#### 3.2.2. Qaternised Chitosan

Chitosan, a naturally-derived polycationic polymer, has been made into various biomedical devices due to its proper biodegradability, good biocompatibility and antimicrobial activity [101,102]. To further extends its application potential, the biological properties of chitosan have been optimized by a series of modifications, one of which is quaternisation. By introducing a permanent positively charged quaternary ammonium group on the dissociative hydroxyl groups or amino groups, quanternized chitosan showed a significantly enhanced antibacterial activity in comparison with the unmodified chitosan in a physiological condition [103,104,105]. Quanternized chitosan could also effectively kill antibiotic-resistant bacteria, such as Methicillin-resistant *Staphylococcus aureus* [106]. The antimicrobial activity of quanternized chitosan was positively correlated with the degree of substitution [104]. However, on the other hand, the higher degree of substitution of quanternized chitosan was also associated with a higher toxicity to osteogenic cells [106].

The mechanisms of the antibacterial activities of quanternized chitosan are still not totally clear. One of the major mechanisms is the clectrostatic interaction between the polycationic groups of quanternized chitosan and the anionic components of microorganisms. This is supported by the fact that quanternized chitosan with a higher degree of substitution exhibited a strong interaction with negative charges on the bacterial cell surface and showed better antibacterial activity than chitosan [107]. Another antibacterial mechanism is theinteractions between the hydrophobic aryl substituents of quanternized chitosan and the hydrophobic interior of bacterial cell walls. This mechanism is supported by the fact that alkyl substituents with an increased chain length on the quanternized chitosan salt also displayed higher antibacterial activities [108]. In addition, the physical states and molecular weight also influence the antibacterial action of quanternized chitosan. In principle, low-molecular-weight quanternized chitosan can penetrate the cell walls of bacteria, thus inhibiting the synthesis of mRNA and DNA transcription [109]. Except for its physicochemical property, the antibacterial potency of quanternized chitosan is also influenced by the property of microorganisms. For example, *Escherichia coli* are less sensitive to quanternized chitosan than *Staphylococcus aureus*, since the outer membrane of Gram-negative bacteria functions as an efficient barrier against chitosan derivatives [110].

## 4. Co-Delivery Systems for Antibacterial and Osteoinductive Drugs to Repair Infected Bone Defects

A proper co-delivery system is of a paramount importance to achieve a successful restoration of infected bone defects. This co-delivery system should first fulfil the general requirements for a proper bone substitute: for example, good biocompatibility, sufficient mechanical strength, high osteoconductivity and proper degradability. Furthermore, the system must also be capable of locally and slowly delivering both antibacterial and osteoinductive drugs. The drugs can be combined with biomaterials either by superficial adsorption, or binding with a chemical bond, or by an internal encapsulation or by a coating layer. Accordingly, the drugs can be released through natural desorption, the enzyme-mediated breaks of the chemical bonds or the degradation of carrying materials. For a co-delivery system, both antibacterial and osteoinductive drugs can be released either in a simultaneous mode or in a sequential mode.

### 4.1. Adsorption and Physicochemical Bonds

**Figure 2:**
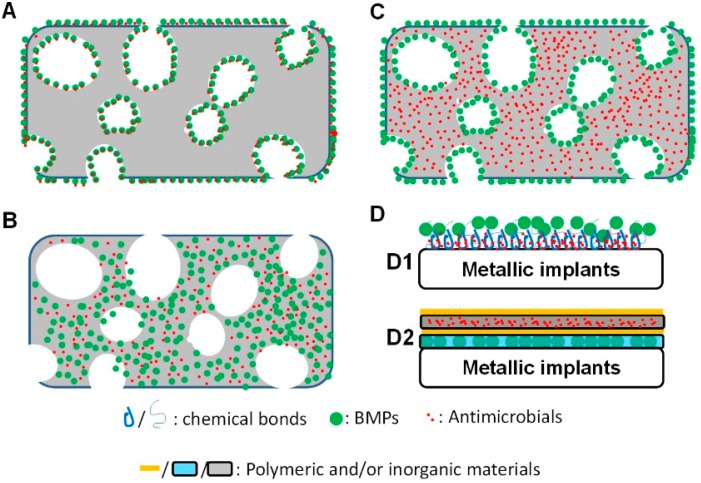
Schematic graph depicting the four different carrying modes of both antibacterial and osteoinductive drugs in biomaterials aiming to treat infected bone defects. (**A**) Superficial adsorption with or without physicochemical bonds; (**B**) A co-encapsulation; (**C**) A mixed carrying mode with encapsulated antibacterial drugs and superficially adsorbed BMPs; (**D**) surface coatings: (**D1**) both drugs are immobilized by chemical bonds; (**D2**) both drugs are encapsulated in coating layers with separation layers for a controlled release.

In clinic, superficial adsorption onto clinically used materials (such as collagen or deproteinized bovine bone) is the most common way to apply bioactive agents (Figure 2A). This is largely due to the hindrance derived from the expensive and time-consuming translational process of medical materials. For example, although BMPs were found in 1965, BMPs can be applied in clinic only through their adsorption onto collagen membrane. In this method, a large portion of BMPs is released in a short time after the expose to the physiological milieu, which will be rapidly deactivated by enzymes in body. Consequently, an unphysiologically high amount (e.g., milligrams) of BMPs has to be applied to elicit the osteoinductive effects [111]. However, the transiently high amount is associated with a series of potential possible side effects, such as an over-stimulation of local bone resorption [54].

On the other hand, the introduction of new materials that bear physicochemical binding sites for drugs may render the release of adsorbed drugs much slower (Figure 2A). Pacheco *et al.* used a silica calcium phosphate nanocomposite as a co-delivery system for vancomycin and BMP2. Using a Fourier transform infrared spectroscopy, the authors showed the interactions between PO_4_^−3^ and negatively charged moieties of vancomycin as well as between the Si–O–Si functional groups and BMP2 [112]. The differences in bonding sites and bonding energy between the ceramic and drugs could confer the co-delivery system significantly different drug release kinetics: vancomycin exhibited a burst release in the first 8 h and thereafter a slow release for up to 28 days. The initial burst release of vancomycin at sufficient but subtoxic concentrations was desirable to prevent an infection in high-energy bone injuries. In contrast, BMP2 showed a slow release without a significant burst. In addition to the physicochemical reactions, the slow release of BMP2 could also be partially attributed to the high resistance of the high-molecular-weight BMP2 to pore diffusion [113].

### 4.2. Co-Encapsulation for a Simultaneous Release

In contrast to the superficial carrying mode, drugs can also be encapsulated into carrying materials (Figure 2B). The release kinetics of encapsulated drugs is mostly simultaneous and is largely dependent on the permeability and the degradability of the carrying materials. An encapsulation requires that the carrying materials can transit from a liquid phase to a solid phase. The simplest way to realize such a phase transition is to remove solvents. Sun *et al.* used collagen as a co-delivery system for AgNPs and BMP2 [114]. They used collagen in aqueous phase to produce AgNPs since the amino acid residues could easily conjugate metal ions via affinity interactions [115]. After the addition of BMPs, the transition from aqueous to solid phase was realized by lyophilization. The BMP2/AgNP/collagen scaffold composites showed a strong antibacterial activity without adversely affecting the adherence or proliferation of bone marrow-derived MSCs (BMSCs). The released BMP2 promoted the osteogenic differentiation of BMSCs, which was symbolized by the upregulation of Runx2, osteopontin and osteonectin expressions [114].

Calcium sulfate, a quick self-setting material, is widely used as a bone-defect-filling material. Furthermore, it is also frequently adopted as an antibiotic carrier for the treatment of infected bone defects [116]. It has many advantages such as low price, full biodegradability, good biocompatibility [117] and high osteoconductivity. Wang *et al.* used calcium sulfate to carry BMP2 and vancomycin through an internal co-encapsulation [31]. In a bone defect in the proximal tibia, the composite significantly augmented new bone formation compared to the control [31]. However, this kind of materials usually forms a solid block and lacks of porous structure, which may hinder the ingrowth of bone tissues. Furthermore, the self-setting process may also compromise the bioactivity of BMP2. In another study, the BMP2 was first adsorbed onto chitosan/calcium phosphate microspheres, which was thereafter embedded into calcium sulfate [118]. This method might largely avoid the influence of self-setting process on BMP2 activity. Unfortunately, the antibacterial and osteoinductive effects of these composites were not further investigated in an *in vivo* infected bone defects.

Polymers are another group of materials that can easily realize a porous structure and the encapsulation of dual drugs. Guelcher *et al.* introduced biodegradable polyurethane (PUR) scaffolds to deliver BMP2 and vancomycin [119]. The solid porous PUR scaffolds were fabricated using lysine triisocyanate, polyester triol, TEGOAMIN33 catalyst and calcium stearate pore opener, *etc.* [120]. Before the transition from the liquid phase to the solid phase, 340 mg/implant vancomycin with or without BMP2 of either a low (2.5 μg/implant) or a high amount (25 μg/implant) was encapsulated in the scaffolds. The vancomycin release kinetics consisted of two phases: (1) the first burst release for a week to protect the graft from contamination and (2) a subsequent sustained release with over the minimum inhibitory concentrations for *Staphylococcus aureus* for 2 months. In an infected rat femoral segmental defect, the dual-delivery composite resulted in substantially more new bone formation and a modest improvement in infection than PUR + BMP2 and collagen + BMP2 treatments [119].

### 4.3. A Mixed Carrying Mode for a Sequential Release

Antibacterial and osteoinductive drugs can also be delivered by a carrying material through different carrying modes. For example, an antibacterial drug is encapsulated into a carrying material with an osteoinductive drug superficially adsorbed onto its surface, or *vice versa*. The two carrying modes can realize different aims: the former mode is mainly aimed for promoting bone regeneration with a prevention of potential infection, while the latter mode is mainly aimed for suppressing an existing bacterial activity and thereafter promoting bone regeneration. Most of the current studies with a mixed carrying mode for BMP2 and antibacterial drugs focused on the former mode (Figure 2C). Although superficial adsorption is not favorable for the maximal osteoinductive efficacy of BMP2, it can largely preserve the bioactivity of BMP2 by avoiding chemical crosslinking during the encapsulation. With this principle, Song *et al.* developed a pHEMA [(poly(2-hydroxyethyl methacrylate)]/nHA (nanocrystalline hydroxyapatite) composite. This composite exhibited a series of positive physicochemical and biological properties, such as good osteoconductivity, an elasticity for surgical press-fitting, and an attractive release profile for proteinaceous drugs [121]. In this composite, nHA was added to enhance the osteoconductivity of the composite. Vancomycin of up to 4.8 wt % could be encapsulated without compromising the structural integrity and compressive modulus of the composite. The encapsulated vancomycin was released in a sustained manner over 2 weeks, which could significantly inhibit the growth of *Escherichia coli*. The BMP2 preabsorbed onto the pHEMA-nHA-vancomycin composite was continuously released over 8 days, which induced osteogenic differentiation of C2C12 cells [30]. In critical rat femoral segmental defects in a latter study, the authors showed that the pHEMA-nHA-vancomycin-BMP2 composites could achieve full bridging with substantially mineralized callus and partial restoration of torsional strength [122]. This carrying material was also used to deliver tetracycline, BMP2/7 heterodimer and RANKL (Receptor activator of nuclear factor kappa-B ligand). A single dose of 40 ng BMP2/7 or 10 ng RANKL from the composite could induce the osteogenic differentiation of myoblast (C2C12 cell line) and the osteoclastogenesis of macrophages (RAW 264.7 cell line) respectively [123].

In a similar principle, Zheng *et al.* constructed a composite containing AgNPs (with a size of 20–40 nm) and PLGA with superficially adsorbed BMP2 [32]. The PLGA/BMP2 composite containing 2.0% AgNPs repaired the *Staphylococcus aureus* Mu50-contaminated femoral defects in 12 weeks without the evidence of residual bacteria. In contrast, 0% or 1.0% AgNPs-PLGA/BMP2/BMP2 composites failed to repair the defects, leaving the presence of continued bacterial colonies. The results indicated that AgNPs of a defined particle diameter exhibited a strong bactericidal effect without a significant cytotoxicity or a compromised osteoinductivity of BMPs. These positive properties made the AgNPs-PLGA/BMP2/BMP2 composite very promising in treating infected bone defects [32].

On the other hand, as abovementioned, the superficially adsorption is less favorable for the osteoinductive efficiency of BMP2. In another study, the authors tried to modify the carrying material to slow down the release of the superficially adsorbed BMP2. Zhou *et al.* used zein, a major starch storage protein found in corn, as a carrying material for antibacterial HACC (hydroxypropyltrimethyl ammonium chloride chitosan) (a quanternized chitosan) and BMP2 [124]. 10 wt % HACC was encapsulated into zein, which showed a strong antibacterial effect without significantly compromising cell proliferation. Different amounts of mesoporous silica SBA-15 nanoparticles were added into zein in order to provide a sustained and localized release of therapeutically relevant factors through their large and highly ordered pores and uniform tunable channels [125]. The release of the superficially adsorbed BMP2 was significantly slowed down with the higher ratio of mesoporous silica SBA-15 nanoparticles. In addition, the mesoporous silica SBA-15 nanoparticles also enhanced the cell viability of human MSCs. In a radial bone defect model (20 mm in length and 5 mm in diameter) in rabbits, zein-HACC-S20-BMP2 composite almost fully repaired and recanalized the bone marrow cavity after 12 weeks. The authors concluded that Silica/HACC/zein scaffolds with both antibacterial and osteoinductive activities had an immense potential in orthopedics and other biomedical applications [126].

### 4.4. Surface Coatings

Over the past decades, metallic implants have been widely used in the fields of orthopedic and dentistry. Although most of them are modified via changing the roughness and hydrophilicity of titanium [127], metallic implants are biologically inert and cannot induce new bone regeneration or inhibit bacterial activity. Such a limitation makes their implantation very challenging when they are used in the sites with compromised bone regeneration capacity and high infection risk, such periodontitic sockets or open bone fractures. In these cases, it is in great need to develop implants with both antibacterial and osteoinductive functions. One approach is to coat the surfaces of implants. Attempts have been done to prepare either polymeric coatings with incorporated antibacterial drugs [128] or inorganic coatings with incorporated BMP2 [129,130]. By carefully combining the principles of polymeric and inorganic coatings, Xie *et al.* developed a co-delivery system for antibacterial and osteoinductive drugs, which contained electrochemically deposited chitosan/Ag/HA and adsorbed haperin/BMP2 [131] (Figure 2(D1)). This coating could simultaneously release Ag^+^ and BMP2. Nevertheless, none of these studies generated sequential releases of several substances. To introduce a co-delivery system with a sequential release, Strobel *et al.* manipulated the concentrations and sequences of one polymer-[poly(d,l-lactide)] (PDLLA), as a sequential drug delivery coating with three distinctly different release profiles: (1) a burst release of gentamicin; (2) a burst release of IGF-I followed by a sustained release and (3) a slow release of BMP2 [124]. Gentamicin, incorporated in the outer layer, exhibited a burst release profile, which was due to the very thin 0.5× or 1× PDLLA layers and its direct expose to an aqueous environment. Thereby, IGF-I (insulin-like growth factor I), in the middle layer, exhibited a fast release and a subsequent slow release, which was controlled by the erosion of the thicker middle layer. BMP2, in the inner layer, exhibited no significant burst release but a slow and sustained release by this sandwich approach [124]. Such a sequential release profile was supposed to exert the functions of these drugs sequentially: the rapidly-released gentamicin to suppress bacterial activities; the secondly-released IGF-1 to stimulate the proliferation of osteoblasts; and the slowly-released BMP2 to enhance an osteogenic differentiation. The following *in vitro* evaluation suggested that the orchestrated delivery of various factors might prevent infections and stimulate bone healing [124].

Using a layer-by-layer (LBL) principle, Min *et al.* introduced a novel system that could realize a tunable staged release of dual drugs for orthopedic implants (Figure 2(D2)) [132]. This multilayered coating consists of two parts: a base osteoinductive component by dipping into a sodium acetate solution containing BMP2, poly(β-amino esters) (*M*n ~ 10 kDa) and poly(acrylic acid) (*M*_W_ ~ 450 kDa); and an overlying antibacterial layer by dipping into a sodium acetate solution containing gentamicin, poly(β-amino esters) (*M*n ~ 11 kDa) and poly(acrylic acid) (*M*_W_ ~ 1.25 MDa). On each layer, polymer/clay barrier layers were deposited using a programmable spray-LBL technique to provide a physical separation of the two components and control interlayer diffusion. Cationic chitosan or poly(diallyldimethylammonium chloride) (*M*_W_ ~ 200–300 kDa) and anionic laponite clay were alternately sprayed onto both osteoinductive and antibacterial layers. The clay barriers could lead to an about 50% reduction in bolus doses and a 10-fold increase in the time span of release. The temporal separation between the release of gentamicin and BMP2 was further enhanced by the laponite barrier, which resulted in a more physiological dosing of BMP2 [132].

## 5. Conclusions

The repair of infected bone defects remains a formidable challenge in the fields of oral implantology, maxillofacial surgery and orthopedics. Due to the less optimal efficacy of current clinical treatments, novel biomaterials with both antibacterial and osteoinductive properties have been developed in order to provide a viable treatment option. In comparison with the clinically used antibiotics, many novel antibacterial biomaterials showed very promising application potential due to their broader bactericidal spectrum, nearly no resistance and good biocompatibility. BMPs, particularly BMP2, are the most potent osteoinductive drugs to induce an *in vitro* osteoblastogenesis and an *in vivo* osteogenesis. The antibacterial and osteoinductive drugs can be incorporated into co-delivery system through the following modes: (A) superficial adsorption/binding with a chemical bond; (B) an internal encapsulation; (C) a mixed carrying mode with a superficial adsorption and an internal encapsulation; and (D) a surface coating (Figure 2). By manipulating the carrying modes, the antibacterial and osteoinductive drugs can be released in varied modes with different kinetics (burst or slow) and temporal characteristics (simultaneous or sequential). These novel biomaterials with both antibacterial and osteoinductive properties showed very a promising potential for clinical applications.
